# Inhospital outcome of elderly patients in an intensive care unit in a Sub-Saharan hospital

**DOI:** 10.1186/s12871-018-0581-x

**Published:** 2018-08-25

**Authors:** M. Lankoandé, P. Bonkoungou, A. Simporé, G. Somda, R. A. F. Kabore

**Affiliations:** 1 Anaesthesia Intensive Care, Regional Hospital of Koudougou, Koudougou, Burkina Faso; 2 Anesthesia Intensive Care, Yalgado Ouedraogo Hospital, Ouagadougou, Burkina Faso; 3 Yalgado Ouedraogo Hospital, Ouagadougou, Burkina Faso; 4 Anesthesia Intensive Care, Blaise Compaoré Hospital, Ouagadougou, Burkina Faso

**Keywords:** Elderly, Intensive care unit, Mortality

## Abstract

**Background:**

In Burkina Faso, demographics are changing and we are seeing a growing prevalence of older patients in intensive care units. Elderly people have increased health care needs but there is a lack of geriatric specialists. This study aimed to analyze in-hospital outcome of patients aged over 65 years, admitted to the Intensive Care Unit (ICU) at Yalgado Hospital.

**Methods:**

We carried out a 5-year retrospective study in the ICU of Yalgado Ouédraogo Hospital. Elderly patients with completed records were included. Baseline characteristics, clinical and outcome were analyzed.

**Results:**

Two thousand one hundred sixteen patients were admitted to ICU, 237 (11.2%) of whom were included. There were 70 females and 167 males. The median age was 71.7 ± 6.1 years. The overall mortality rate in ICU was 73%, of whom 90% died within 7 days after admission. In multivariate analysis, shock (Odds Ratio: OR = 2.2, *p* = 0.002), severe brain trauma (OR; 9.6, *p* = 0.002), coma (OR 5.8 *p* < 0.003), surgical condition (OR = 4.2, *p* = 0.003), ASAPS Score ≥ 8 (OR = 4.3, *p* = 0.001), complication occurring (OR = 5.2_,_ *p* = 0.001) and stroke (OR = 3.7_,_ *p* = 0.001) were independent factors.

**Conclusion:**

Elderly patients were frequent in ICU and their mortality rate was high. Stroke, severe brain trauma, surgery, complications occurring during hospitalization were independent risk factors of death.

## Background

Elderly is defined as a chronological age equal to or above 65 years worldwide or above 60 years in Africa [1]. Worldwide, the elderly population continues to grow due to increased life expectancy [2]. In 2015, the world elderly population rose by 55 million and the proportion reached 8.5% [3]. In Africa, the proportion of elderly accounted for 6.6% in 2015 and will reach 9.6% in 2050. This increase is also seen in Burkina Faso. In 1985, the census in Burkina Faso reported 319,496 elderly people accounting for 4% of the general population. [4] This rose to 475,812 (2.4%) in 2016 [5]. Despite the increase of the number of elderly people in Burkina Faso, their proportion is reduced by the explosion of births and the youth of the population. One of the consequences of the growth in the number of elderly patients is the increased requirement for admission to ICU. They require appropriate healthcare facilities, and special skills and human resources. They have higher morbidity and mortality because of associated co-morbidities. In developing countries, intensive care is limited by poverty, lack of equipment, inadequate skills and insufficient human resources. This study aims to assess outcomes for elderly patients admitted to ICU in the first referral teaching hospital Yalgado Ouédraogo in Burkina Faso.

## Methods

We carried out a retrospective study of elderly patients admitted to the ICU of the tertiary Hospital Yalgado Ouédraogo over a five-year period (1st January, 2011 to 31st December, 2015). Yalgado Hospital is a tertiary care, governmental hospital, with an overall capacity of 800 beds and an 8 bed ICU where patients are managed by anesthesiologists. The average number of admissions to ICU was about 200 patients per year with a mortality of 51.6% [6]. The ICU is poorly equipped (one transport ventilator, one defibrillator and monitors). Patients receive fluid and electrolyte management, transfusions, oxygen, vasoactive drugs, and nutrition (parenteral and oral nutrition). Non-invasive ventilation and CPAP were not used. Relevant data recorded includes socio-demographic characteristics, co-morbidities, diagnosis, indications for ICU admission, Glasgow Coma Score at admission, the Ambulatory Simplified Acute Physiologic Score (ASAPS) [7], Charlson co-morbidity score, need for mechanical ventilation, blood transfusion and or hemodialysis, sepsis, shock on admission, length of stay and outcome. The Ambulatory Simplified Acute Physiologic Score is a scale used for patients admitted to ICU to evaluate the severity of their condition.

Ethical and National Scientific Research and Technology Center (ENSRTC) approved the study. The Epidemiologic Information package version 7.1.5.0 was used for data analysis. Descriptive statistics included frequency for nominal variables and mean ± standard deviations or medians and interquartile ranges for continuous variables according to their distribution. Independent *t***-**test (continuous variables) and Chi-square test (categorical variables) were used in univariate analysis when comparing age groups, survivors to non-survival patients. A *p* value ***≤***0.05 was considered as significant. The total sample was divided into three groups according to age (65–74 years or « young old », 75–84 years or old old, and > 85 years or oldest old).

## Results

In total, 2116 patients were admitted to ICU of whom 237 (11.2%) were included in our study. The mean age was 71.7 ± 6.1 years, with male: female ratio of 2.3. The demographic characteristics are summarized in Table 1.

**Table 1:** Demographic characteristics of patients (*n* = 237)

Characteristic	Mean	Number	Percentages
Age (years)	71.7 ± 6.1		
65–74 years		167	70.5
75–84 years		58	24.5
Over 84 years		12	5
Gender			
Male		167	70.5
Female		70	29.5
Residency			
Urban		159	67.1
Rural area		78	32.9
Profession			
Retired		48	32.6
Housewife		37	25.2
Farmer		31	21.1
Public/Private service		31	21.1
Referral facilities			
District hospital		107	45.1
Regional hospital		39	16.4
Dispensary		3	1.3
Teaching hospital		19	8
Private Hospital		69	29.1

Teaching Hospital (YO: 8 cases; Blaise Compaore Hospital: 6 cases; Sourou Sanou Hospital = 5 cases);

Comorbidity was identified in 191 cases (80.6%) of which 49.4% (*n* = 117) had more than 2 comorbidities and 19.4% (*n* = 46) had none. The Charlson median score was 4.8. A score ≥ 4 was recorded in 89.4% at admission in ICU. Past histories of hypertension (50.6%), diabetes (23.6%) and peptic ulcers (6.3%) were common. The clinical features are summarized in Table 2. 42.1% of patients were comatose, with Glasgow coma score < 8. 100 (49.02%) patients had ASAPS score ≥ 8.

**Table 2:** Clinical characteristics of patients (*n* = 237)

Clinical characteristics	Mean	Number	Percentage
Reasons of admission (*n* = 237)			
ACS^a^		133	56.1
Shock		78	32.9
Thermal burns		11	4.6
ARDS^b^		9	3.8
Poor condition		6	2.5
Glasgow coma score (*n* = 214**)**	9.6 ± 4.0		
< 8		90	42.1
8–14		78	36.4
15		46	21.5
Blood Pressure (*n* = 237)			
Systolic Pressure	132.5 ± 36.6		
Diastolic pressure	78.1 ± 23.5		
Hypertension		124	52.3
Hypotension		35	14.7
Temperature (*n* = 237)		111	46.8
Hyperthermia			
Hypothermia		9	3.8
ASAPS (*n* = 204)	7.9 ± 3.5		

*ASAPS* Ambulatory simplified acute physiologic score

^a^*ACS* Alteration of consciousness

^b^*ARD* Acute Respiratory Distress Syndrome

Medical conditions (60%), particularly of the Central Nervous System (CNS) (37.97%), were the most common. Among all diseases, stroke was most frequent (27.4%) followed by peritonitis. Care was based on fluid and electrolyte management, pain relief, and supply of oxygen. Only 2 patients were mechanically ventilated. During hospitalization, complications occurred in 89 patients (37.55%) of which acute respiratory distress syndrome (ARDS) was the main one (10.55%). In total 173 (73%) patients died in ICU. Table 3 summarizes the diagnoses and in-hospital outcome of patients.

**Table 3:** Diagnosis and outcome of patients (*n* = 237)

Clinical data	Number	Percentage
Admission condition		
Medical condition	183	77.2
Surgical condition	54	22.8
Diseases		
Stroke	65	27.4
Prostate tumor	27	11.4
Sepsis	26	10.9
Trauma/Burn	25	10.5
Bowel obstruction	13	5.5
Heart disease	18	2.5
Diabetes Acute metabolic complications	20	8.4
Kidney failure	16	6.7
Other^a^	27	11.4
Total	237	100
Complications		
Sepsis	25	10.5
Acute Respiratory Distress Syndrome	38	42.7
Shock	15	6.3
Coma	19	21.3
Bed sores	8	3.4
Acute pulmonary edema	5	2.1
Pulmonary aspiration	5	2.1
Pulmonary embolism	1	0.4
Other^b^	5	2.1
Outcomes		
Death in ICU	173	73
Transfer to other ward	48	20.2
Hospital discharge with physician authorization	10	4.2
Discharge without physician authorization	6	2.5
Total	237	100

^a^Other: anemia (*n* = 3), dehydratation (*n* = 2)

^b^Hernia, blood disorder, ulcer, hydronephrosis, asthma, skin disease, leukemia

The mean length of ICU stay was 5.3 ± 7.4 days [IC 95%; 1–58]. Ninety percent of those that died, did so within a week while 10% died after a week. Survivors and non-survivors were comparable based on demographic data (Table 4). When comparing survivors to non-survivors, there was a significant difference related to the emergency context (*p* = 0.001), surgical condition (*p* = 0.003**),** coma condition (*p* = 0.001**),** shock (*p* = 0.002**)**, Charlson score **≥** 8 (*p* = 0.03), ASAPS score 8 ≥ at admission (*p* = 0.0001), stroke (*p* < 0.0001), diabetic complication (*p* = 0.01**)**, and complication in ICU (*p* = 0.001) in univariate analysis.

**Table 4:** Comparison of survivors and non-survivor’s patients (*n* = 237)

Characteristics	All patients (*N* = 237)	Non-survivors (*n* = 173)	Survivors (*n* = 64)	*p* value
Age (Mean; years)	71.7 ± 6.1	71.6 ± 5.9	72.1 ± 6.4	0.5
Age group (%)				
65–74	167 (70.4)	123 (73.6)	44 (26.3)	0.7
75–84	58 (24.5)	42 (72.4)	16 (27.6)	0.9
85 above	12 (5.1)	8 (66.6)	4 (33.3)	0.6
Gender				
Male (*n* = 167)	167 (70.4)	127 (76.05)	40 (23.9)	0.1
Female (*n* = 70)	70 (29.6)	46 (65.7)	24 (34.3)	
Reference specialty (%)				
Emergency service	134 (56.4)	108 (80.6)	26 (20.4)	0.001
Medicine	21 (8.8)	14 (66.6)	7(33.8)	0.4
Surgery	54 (22.8)	49 (90.7)	5(9.3)	0.003
Reasons for admission				
ACS	133 (56.1)	109 (81.9)	24 (18)	< 0.001
Poor condition	6 (2.5)	3 (50)	3 (50)	0.3
Burn	11 (4.6)	7 (63.64)	4 (36.4)	0.4
ARDS	9 (3.8)	7 (77.7)	2 (22.2)	1
Shock	78 (32.9)	47 (60.3)	31 (39.7)	0.002
Charlson Score (Median)	4.8 ± 1.8	4.6 ± 1.7	5.09 ± 2.04	0.12
≥ 8	20 (8.4)	11 (55)	9 (45)	0.03
< 8	217 (91.6)	103 (47.4)	76 (52.6)	0.4
Glasgow coma score (Mean valu)	9.64 ± 4.01	8.9 ± 3.8	11.8 ± 3.6	0.03
< 8	42.06	69 (88.4)	9 (11.5)	< 0.001
≥ 8	57.9	58 (68.2)	76 (31.8)	
ASAPS score (Mean)	7.9 ± 3.5	8.6 ± 3.5	5.8 ± 2.6	< 0.001
ASAPS ≥8		150 (87)	13	
ASAPS < 8		109 (63.5)	36.6	
Diagnosis				
Stroke	65 (27.4%)	57 (87.69%)	8 (12.31%)	0.001
Peritonitis	22 (9.3%)	18 (81.82%)	4 (18.18%)	0.4
AMCD^A^	20 (8.4%)	10 (50%)	10 (50%)	0.01
SBT^B^	19 (8%)	18 (94.74%)	1 (5.26%)	0.02
Bowel obstruction	13 (5.5%)	7 (53.85%)	6 (46.15%)	0.1
Burn	10 (4.2%)	6 (60%)	4 (40%)	0.4
Severe infection	10 (4.2%)	8 (80%)	2 (20%)	0.7
Prostatic adenoma	9 (3.8%)	5 (55.56)	4 (44.44)	0.2
Heart disease	6 (2.5%)	4 (66.67%)	2 (33.33%)	0.6
Prostatic cancer	5 (2.1%)	1 (20%)	4 (80%)	0.01
Inguinal hernia	5 (2.1%)	3 (60%)	2 (40%)	0.6
Other^C^	53 (22.4%)	36 (67.92%)	17 (32.08%)	0.1
Complications occurred in ICU				
Yes = 89	89 (37.5%)	80 (89.9%)	9 (10.1%)	< 0.001
Non = 148	148 (62.5%)	93 (62.8%)	55 (37.2%)	
Mechanical ventilation	2 (0.8%)	2 (100%)	0	Ki = 0.7
Length of stay in ICU (mean)	5.3 ± 7.4	5.2 ± 8	5.5 ± 5.1	0.8

*ACS* Alteration of consciousness, *ARDS* Acute Respiratory Distress Syndrom, *AMCD* Acute metabolic complication of diabetes, *SBT* Severe brain trauma

*ICU* Intensive Care Unit, *ASAPS* Ambulatory Simplified Acute Physiologic Score

^A^ Acute Metabolic Complication of Diabetes

^B^ Severe Brain Trauuma

^C^ Other disease

Most patients were between 64 to 74 years old. There was a significant difference between the age groups for Charlson score (*p* = 0.001) and complications in ICU (*p* = 0.01) (Table 5).

**Table 5:** Comparison of patients according to age group (*n* = 237)

Variables	65–74 years *n* = 167 (70.4%)	75–84 years *n* = 58 (24.5%)	Over 84 years *n* = 12 (5.1%)	*p* value
Age (Mean; years)	68.3 ± 2.8	78.2 ± 2.5	86.6 ± 1.6	< 0.001
Gender				
Male (*n* = 167)	118	40	9	0.9
Female (*n* = 70)	49	18	3	
Reasons for admission				
ACS	92	33	6	0.9
Poor condition	68	26	5	0.6
Burn	7	2	1	0.7
ARDS	13	6	0	0.4
Shock	11	4	2	0.4
Charlson Score (Median)	4.5	5.1	6.3	0.001
≥ 8	12	5	3	0.1
< 8	155	53	9	
Glasgow coma score (mean)	9.7 ± 4.02	9.4 ± 4.1	8.8 ± 3.5	0.6
< 8	12	5	3	0.1
≥ 8	155	53	9	
ASAPS score (Mean)	7.9 ± 3.8	8.08 ± 2.9	8.1 ± 2.7	0.9
ASAPS ≥8	65	25	8	0.1
ASAPS < 8	77	22	4	0.4
Complications in ICU				0.01
Yes = 89	65	19	5	0.6
Non = 148	102	39	7	
Mechanical ventilation	2	0	0	0.6
Length of stay ICU (mean)	5.3 ± 6.8	5.6 ± 9.2	2.7 ± 2.2	0.4
Death	123 (73.6)	42 (72.4)	8 (66.6)	0.2

*ACS* Alteration of consciousness, *ARDS* Acute Respiratory Distress Syndrom, *AMCD*, Acute metabolic complication of diabetes, *SBT* Severe brain trauma

In multivariate analysis, surgery, coma at admission, shock, stroke, and severe brain trauma were independent risks factors of ICU death (Table 6).

**Table 6:** Risk factors for ICU mortality of elderly patients

Diagnosis	Adjusted OR (CI 95%)	*p*
Clinical situation	Reference	
Surgery	4.2 [2.4–10.3]	0.003
Coma at admission	2.9 [1.6–5.4]	0.001
Coma in ICU	5.8 [2.3–14.6]	
Shock during admission	2.2 [1.6–4.0]	0.002
ASAPS ≥8	4.3 [1.1–8.5]	0.001
Stroke	3.7 [1.6–8.7]	0.001
Severe brain trauma	9.6 [1.2–75.1]	0.02
Complications occurred in ICU		0.001
No	Reference	
Yes	5.2 [2.4–11.3]	0.001

## Discussion

This retrospective study found that elderly patients represented 11.2% of admissions to ICU. This rate is comparable to the (10%) of Owojuyigbe et al. [8] and the (16.6%) of Belayachi et al. [9]. In developed countries elderly admissions rate to ICU was high. The rate of admission in our study may be due to life expectancy shortness that reflects Burkinabè demographic profile.

Patients mean age was 71.7 ± 6.1 years in our study. Other study found a mean age of 72 years in Morocco [9], 75.4 ± 6.8 years in Brasilia [10] and 73 years in Nigeria [8]. In our study, patients were mainly males. There was no correlation between gender and admission in the literature [8, 9]. Some studies reported similar results to ours [11, 12], but Fowler et al. [13] reported higher mortality in the female group.

Patient outcome was poor in our study with a mortality rate of 73%. Belayachi et al. [9] in Morocco, Wade et al. [14] in Senegal reported 44.7 and 42.8% respectively. The high mortality reported in Africa compared to developed countries may be due to inadequate equipment, resources and care limitations [9].

Advanced age alone does not preclude successful outcome [15]. In multivariate analysis independent risk factors were surgical conditions, coma, shock during admission, ASAPS ≥8, stroke, severe brain trauma. This result is comparable to literature reports [16, 17]. In our findings, stroke was the main cause of hospitalization, followed by peritonitis. There is no difference between age groups in term of mortality. The mean length of stay in ICU was 5.3 ± 6.8 days. While 46.6% of patients died within the first 3 days of admission, 90% of patient died within a week. For patients over 84 years, LOS was shorter and in-hospital mortality was less than patients aged less than 84 years. This can be explained by the family taking their relative home once they understand that the outlook for recovery is bleak. This explains the relatively low mortality rate and short stay. The overall poor outcomes may be due to late consultation and poor quality of care due to the inadequate facilities, equipment and lack of medications. Delay in consultation may be related to limited education, use of traditional medicine, poverty with concern about hospital costs and poor transportation.

This study has limitations due to being retrospective. We did not assess the impact of pre-hospitalization condition. Blood test abnormalities, and the impact of APACHE and SOFA scores on outcome were not evaluated and long-term mortality after hospital discharge was not studied.

## Conclusion

These results show that elderly patients in ICU have a high risk of ICU death. Risk factors include coma at admission, shock state, high ASAPS, severe brain trauma, stroke and surgical condition. We need to better equip our ICU to assess and manage carefully elderly patients in order to reduce ICU mortality. Training geriatric specialists could improve chronic disease care of older patients and reduce their admission to ICU. A prospective study could give more information on risk and long term outcome of these frail patients.

## Abbreviations

ACS: Alteration of consciousness
AMCD: Acute metabolic complication of diabetes
APACHE: Acute physiology and chronic health evaluation
ARDS: Acute respiratory distress syndrome
ASAPS: Ambulatory simplified acute physiologic score
ICU: Intensive care unit
NCSRT: National Center of Scientific Research and Technology
OR: Odd ratio
SBT: Severe brain trauma
SOFA: Sequential organ failure assessment
